# Incidence trend of type 2 diabetes from 2012 to 2021 in Germany: an analysis of health claims data of 11 million statutorily insured people

**DOI:** 10.1007/s00125-024-06113-8

**Published:** 2024-02-26

**Authors:** Carolin T. Lehner, Marian Eberl, Ewan Donnachie, Luana F. Tanaka, Gunther Schauberger, Florian Schederecker, Sebastian Himmler, Leonie Sundmacher, Stefanie J. Klug

**Affiliations:** 1 Chair of Epidemiology, School of Medicine and Health, Technical University of Munich, Munich, Germany; 2 Bavarian Association of Statutory Health Insurance Physicians, Munich, Germany; 3 Chair of Health Economics, School of Medicine and Health, Technical University of Munich, Munich, Germany

**Keywords:** Epidemiology, Health claims data, Incidence, Trends, Type 2 diabetes

## Abstract

**Aims/hypothesis:**

The aim of the study is to describe the time trend of type 2 diabetes incidence in the largest state of Germany, Bavaria, from 2012 to 2021, and to compare the incidence rates during the pandemic period (2020–2021) to the pre-pandemic period (2012–2019).

**Methods:**

This secondary data analysis uses health claims data provided by the Bavarian Association of Statutory Health Insurance Physicians (KVB), covering approximately 11 million insurees, accounting for 85% of the total population of Bavaria, Germany. Newly diagnosed type 2 diabetes cases in adults (≥20 years) coded as E11 (Diabetes mellitus, Type 2) or E14 (Unspecified diabetes mellitus) under ICD-10, German modification (ICD-10-GM) for the study period 2012 to 2021 were included. Annual and quarterly age-standardised incidence rates (ASIR) stratified by sex, age and region were calculated using the European standard population. Sex-specific crude incidence rates (CIR) were calculated using 10-year age groups. Regression analyses adjusted for time trends, seasonal effects, and pandemic effects were used to analyse the incidence trend and to assess the effect of the pandemic.

**Results:**

Overall, 745,861 new cases of type 2 diabetes were diagnosed between 2012 and 2021: 50.4% (376,193 cases) in women. The male/female ratio remained stable over the observation period, while the median age at diagnosis decreased from 62 to 59 years in men and from 66 years to 61 years in women. ASIR were consistently higher for men compared with women, with the yearly difference remaining stable over time (2012: 18%; 2021: 20%). An overall decreasing trend in ASIR was observed during the study period, with a strong decrease from 2012 to 2017, followed by a less pronounced decline from 2018 to 2021 for both sexes. For men, ASIR decreased from 1514 per 100,000 person-years in 2012 to 995 per 100,000 person-years in 2021 (4.6% average annual reduction), and for women from 1238 per 100,000 person-years in 2012 to 796 per 100,000 person-years in 2021 (4.8% average annual reduction). This downward trend was also observed for age groups above 50 years. Regression analyses showed no significant change in incidence rates during the pandemic period (2020 and 2021) compared with the pre-pandemic period.

**Conclusions/interpretation:**

For the first time, a 10-year incidence trend of type 2 diabetes is reported for Germany, showing a strong decline from 2012 to 2017, followed by a less pronounced decline from 2018 to 2021. The incidence trend of type 2 diabetes appears not to have been affected by the first 2 years of the COVID-19 pandemic. Despite an overall increasing prevalence, the incidence is decreasing, potentially resulting from robust screening by family physicians, reducing the median age at diagnosis by 3 to 5 years. However, further investigation is needed to fully identify the reasons for the declining incidence trend. Continued incidence monitoring is necessary to identify the long-term trend and the potential effect of the pandemic on diagnoses of type 2 diabetes.

**Graphical Abstract:**

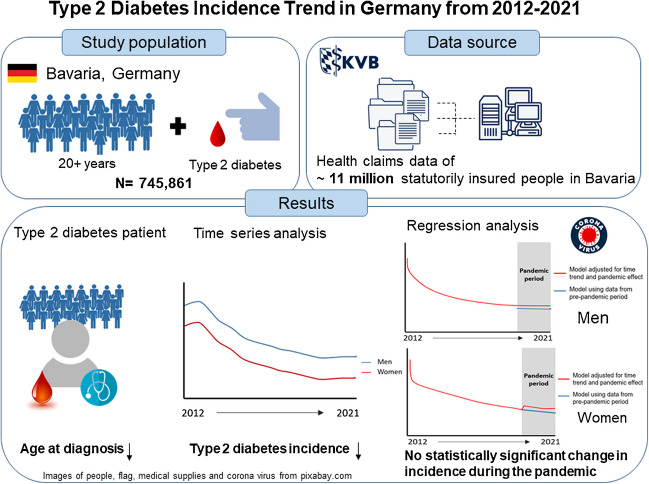

**Supplementary Information:**

The online version contains peer-reviewed but unedited supplementary material available at 10.1007/s00125-024-06113-8.


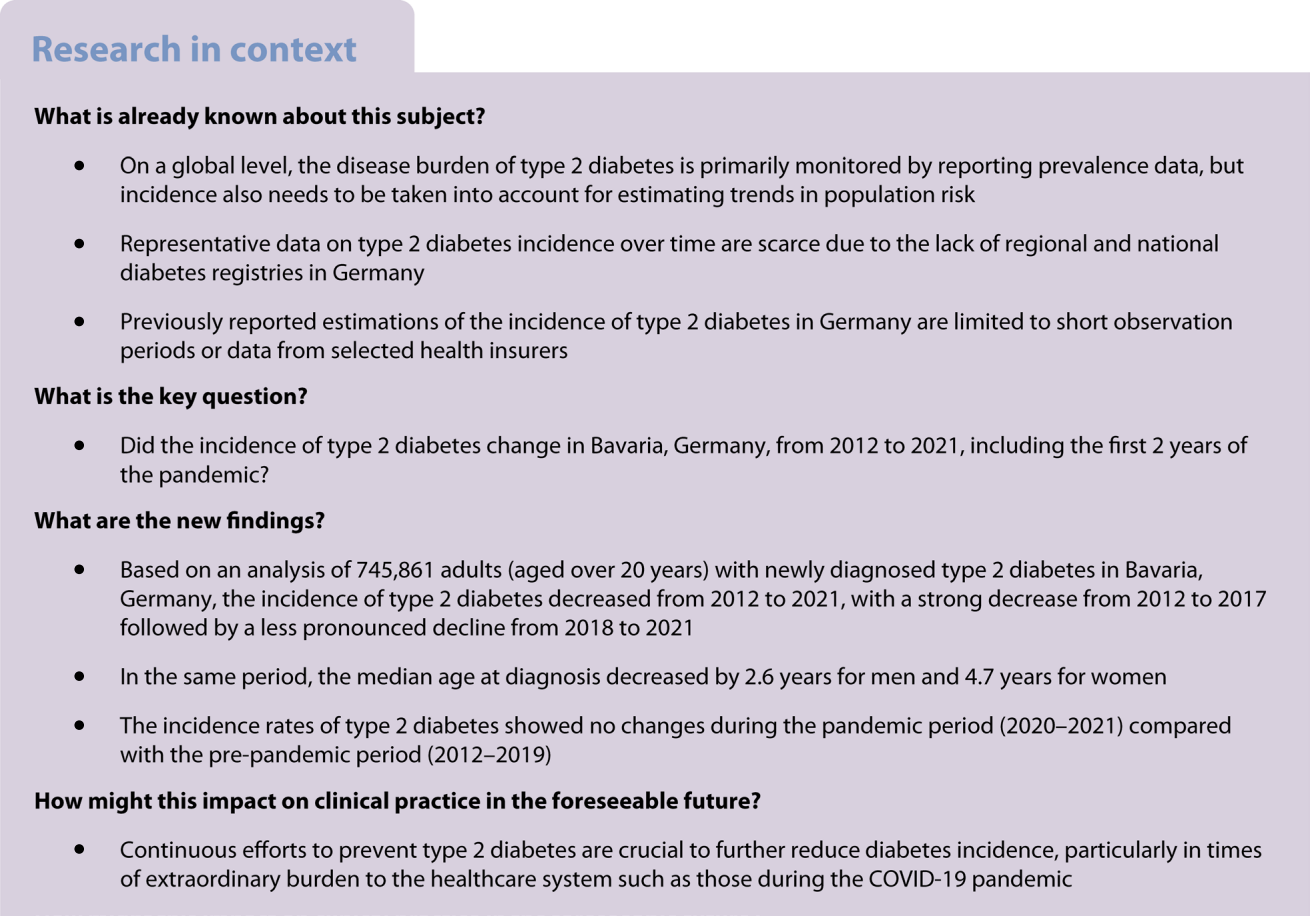


## Introduction

Diabetes mellitus is a significant global health crisis with a high burden on the population and healthcare systems. Approximately 11% of the world population (537 million people) aged 20–79 years were living with diabetes (type 1 or type 2 diabetes) in 2021 [1]. This number is expected to increase to 783 million by 2045 [1]. Over the past decade, the number of people living with diabetes (type 1 or type 2 diabetes) aged 20–79 years increased by 62% globally, with 90% of cases being attributed to type 2 diabetes [1, 2]. In Germany, current estimates of diabetes incidence and prevalence (type 1 or type 2 diabetes) are 1.2% for all age groups and 7.2% for the age group 18–79 years respectively [3]. Predictions for Germany indicate that prevalence of type 2 diabetes will increase by 54% to 77% by 2040 (in people aged 18 and over) [4]. Due to the increased mortality and morbidity, type 2 diabetes imposes a high disease burden in Germany [5]. Sixteen per cent of all deaths in Germany are attributed to type 2 diabetes, with the highest excess in deaths occurring in those aged 70–89 years [5]. Healthcare-related direct costs are 1.7 times higher for people living with type 2 diabetes compared to people without diabetes, with increasing direct costs related to cardiovascular complications, type of treatment (insulin vs other glucose-lowering drugs) and disease duration (>20 years) [6].

Monitoring diabetes trends is crucial for diabetes management and healthcare policy. In Germany, the European Union’s most populous country, reliable data about diabetes incidence and its regional distribution are scarce due to a lack of effective national and federal diabetes registries. Worldwide, only 12 diabetes registries exist, mainly from the northern part of Europe, with variable registry quality. However, not all of them report HbA_1c_ measurements, one of the most important values of glycaemic control [7]. On a global level, incidence rates of type 2 diabetes are rarely published, while reporting on the prevalence of diabetes is a standard measure for both types of diabetes [1]. However, monitoring prevalence alone is not sufficient to measure the disease burden and the effects of prevention programmes because the life expectancy and survival of those diagnosed with diabetes drive prevalence alongside the occurrence of new cases [1]. While increasing prevalence trends of type 2 diabetes in Germany suggest a rise in disease burden, incidence data needs to be taken into account [4] for clinical decisions and health system planning. The few previous reports from Germany on type 2 diabetes incidence are limited to short observation periods or use data from selected health insurance companies, not allowing for comparison with other European countries [3]. Generalisability is hindered due to the smaller samples and differences in the socio-demographics of the insured individuals [8].

Hence, this study aims to describe the time trend of type 2 diabetes incidence in Bavaria, the largest German state, from 2012 to 2021. We also compare the incidence rate during the pandemic period (2020–2021) to the pre-pandemic period (2012–2019) using routinely collected health claims data from all statutorily insured people in Bavaria, Germany.

## Methods

### Data source

This secondary data analysis was conducted using routinely collected health claims data from the Bavarian Association of Statutory Health Insurance Physicians (KVB). Bavaria is the largest and second most populous federal state in Germany, with about 13.2 million inhabitants. The KVB data covers outpatient care of the whole statutorily insured Bavarian population of 11 million people, corresponding to 85% of the regional population. Therefore, if they are eligible for reimbursement, all patient visits, medical events that are reported to the outpatient facility and medical procedures performed, including primary and specialist/secondary care, are health claims data and can potentially trigger a diagnosis code. The KVB collects data quarterly and stores all health claims data from outpatient services with reimbursement eligibility. To ensure patient privacy, the database allocates a unique identification number for each patient, removing all personal information. The data provided by the KVB have been checked for plausibility according to the rules of the claims schedule. Patients were allocated a stable pseudonym based on their (life-long) insurance number, name and date of birth. If pseudonyms consist of multiple names or date of birth that change over time, they are considered implausible and are therefore excluded from the dataset by the KVB prior to data delivery. All analyses were based on anonymised health claims data containing aggregated information about sex, age, year of diagnosis, quarter of diagnosis, regional district, number of incident cases, population at risk and crude incidence rate. Study data were aggregated by 96 regional districts, sex, quarter and 5-year age groups. For the calculation of the population at risk, the official sex- and age-specific number of statutorily insured people in Bavaria aged over 20 years provided by the German Federal Ministry of Health was used [9], excluding prevalent cases to avoid underestimation of the incidence rates. Further, the number of statutorily insured people within the regional districts was derived from the regional distribution of patients' districts of residence observed in the KVB claims data.

This study was approved by the institutional ethical review board of the Technical University of Munich (TUM) School of Medicine (741/21 S) and by the regulatory authority of the KVB, the ‘Bavarian State Ministry of Health and Care’. This study was conducted in accordance with the ‘Good Practice of Secondary Data Analysis’ guideline [10].

### Study population

The study population consists of all cases of newly diagnosed type 2 diabetes in adults aged 20 years and older, coded as E11 (Diabetes mellitus, Type 2) or E14 (Unspecified diabetes mellitus) according to International Statistical Classification of Disease, 10th revision, German Modification (ICD-10-GM) [11], who resided in Bavaria at the time of diagnosis during the study period 2012 to 2021. Only confirmed new diagnoses with the same ICD code in at least two insurance quarters within a maximum of four subsequent quarters (between 2012 and end of the diagnosis period) were included. Patients with a first appearance in the KVB dataset and an incident diagnosis before the observation period were counted as prevalent cases. To further differentiate incidence from prevalent diagnosis for patients newly appearing in the KVB data due to e.g. migration to Bavaria, the cases were counted as prevalent if the quarter of the diagnosis was the same as the quarter of the first appearance in the KVB dataset. Our study covers 10 observation years, with the pre-pandemic period (2012–2019) and the pandemic period (2020–2021).

### Statistical analysis

For the descriptive analyses, crude incidence rates (CIR), age-standardised incidence rates (ASIR), population size and median age at diagnosis stratified by sex and year from 2012 to 2021 were calculated. Annual and quarterly ASIR per 100,000 person-years were calculated with the direct standardisation method, using the European standard population from 2013 as a reference [12]. Sex-specific CIR per 100,000 person-years were calculated with 10-year age groups. Time series plots of ASIR stratified by sex and CIR stratified by eight 10-year age groups (20–29, 30–39, 40–49, 50–59, 60–69, 70–79, 80–89 and 90–120) were used to describe the development of incidence over time. The average annual change in the percentage of incidence was calculated using the geometric mean of the annual growth rate. An interrupted time series regression analysis was used to quantify the incidence trend over time and assess a possible pandemic effect by using three different prediction models for ASIR per 100,000 person-years [13]. The following three regression models were used: The full Model 1, including pandemic and seasonal effects, was used to model quarterly ASIRs adjusted for time trends (change in outcome associated with a time unit increase), seasonal effects (yearly quarters one to three in comparison with quarter 4) and pandemic effects (level/step change of the ASIR trend in the pandemic period compared with the pre-pandemic period); Model 2, with pandemic effects but without seasonal effects, was used to model quarterly ASIR adjusted for time trends and pandemic effects; and lastly, Model 3, without pandemic effects and without seasonal effects, used data from the pre-pandemic period 2012–2019 to predict quarterly ASIR in the pandemic period (counterfactual prediction) [13]. These predictions attempted to predict the ASIR in the absence of a pandemic. The independent variable time was log-transformed to achieve a linear association between ASIR and log time. Therefore, the interpretation of the effect estimations is not straightforward. Unstandardised regression coefficient and 95% CI were calculated. Further, sensitivity analyses were performed using Model 4 without a pandemic effect but with seasonal effects in order to predict quarterly ASIR in the pandemic period, based on the estimation of the pre-pandemic trend of ASIR (counterfactual prediction with seasonal effects). To account for possible misclassification of type 2 diabetes E11 ‘Diabetes mellitus, Type 2’ and E14 ‘Unspecified diabetes mellitus’, sensitivity analyses of ASIR, sex-specific CIR, and regression were conducted only with cases coded as E11 (ICD-10-GM).

All analyses were conducted in R [14], using the tidyverse framework for data management and the ggplot2 package for creating graphics [15, 16].

## Results

### Characteristics of the study population

Overall, 745,861 new cases of type 2 diabetes were diagnosed between 2012 and 2021; 50.4% (376,193 cases) in women and 49.6% (369,668 cases) in men. The median age of diagnosis across the study period was 63.32 years, with a higher age at diagnosis for women (65.58 years) compared with men (61.36 years). The male/female ratio of individuals with type 2 diabetes remained stable over the observation period, while the median age at diagnosis decreased from 61.6 to 59.1 years in men (−2.6 years) and from 66.1 years to 61.4 years in women (−4.7 years). Further characteristics of the study population are presented in Table 1.

**Table 1:** Demographics of the study population aged 20 years and older

	Male					Female				
Year	New cases (*n*)	Mean population at risk (*n*)	CIR^a^	ASIR^b^	Median age (years)	New Cases (*n*)	Mean population at risk (*n*)	CIR^a^	ASIR^b^	Median age, (years)
2012	45,665	3,447,748	1324	1514	61.62	49,214	4,125,290	1192	1238	66.08
2013	43,434	3,481,866	1247	1418	61.32	47,187	4,142,374	1139	1179	65.14
2014	38,067	3,542,583	1074	1215	60.44	40,121	4,171,021	961	993	64.32
2015	35,999	3,592,838	1001	1132	62.48	36,724	4,195,541	875	903	63.55
2016	34,349	3,664,069	937	1057	59.42	34,599	4,234,879	817	841	62.86
2017	33,849	3,748,788	902	1016	59.17	32,846	4,277,835	767	791	62.60
2018	33,834	3,814,124	887	995	59.33	33,181	4,316,388	768	787	62.34
2019	35,354	3,827,483	923	1024	59.41	34,453	4,338,962	794	806	62.10
2020	33,599	3,862,386	869	954	59.16	33,186	4,366,781	759	769	61.54
2021	35,518	3,877,976	915	995	59.06	34,682	4,380,246	791	796	61.39
Total	369,668			1132	61.36	376,193			911	65.58

^a^ CIR, crude incidence rate per 100,000 person-years

^b^ ASIR, age-standardised incidence rate per 100,000 person-years using European Standard Population 2013

New cases, new incident cases of type 2 diabetes; Median age, median age at diagnosis

### Trends in ASIR and CIR of type 2 diabetes

**Fig. 1:**
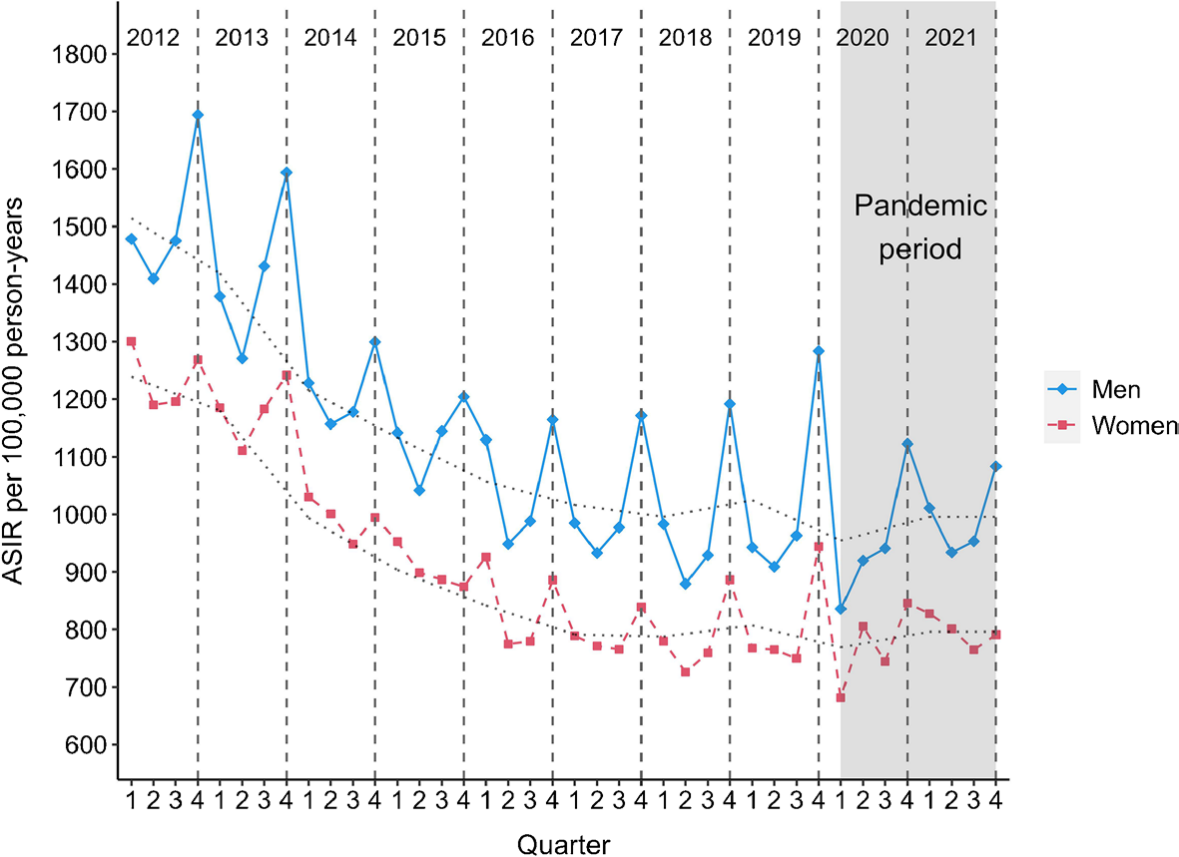
Trends of quarterly ASIR of type 2 diabetes from 2012 to 2021 stratified by sex. Grey shaded area, pandemic period; dotted grey line, yearly ASIR

**Fig. 2:**
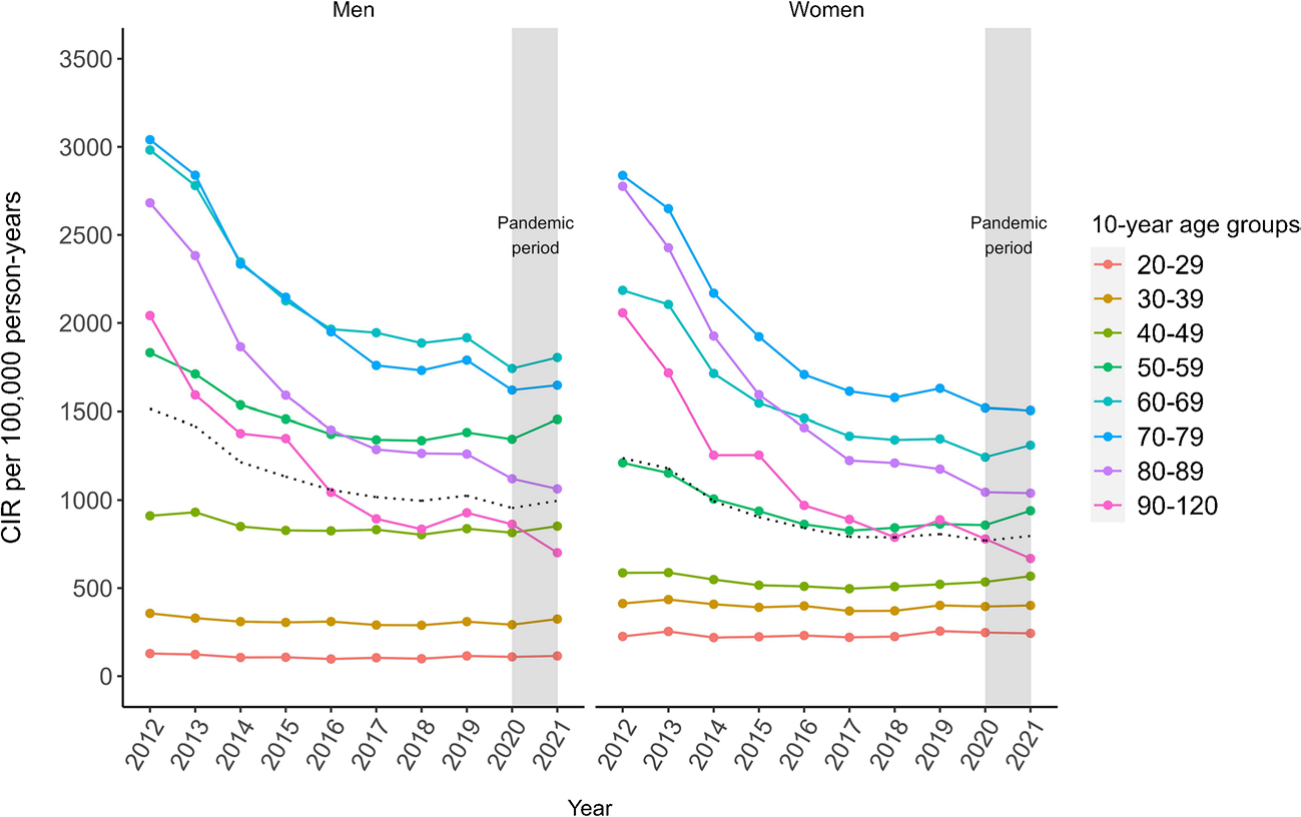
Trends of yearly sex- and age-stratified CIR of type 2 diabetes incidence from 2012 to 2021. Grey shaded area, pandemic period; dotted black line, yearly ASIR

The overall ASIR of type 2 diabetes from 2012 to 2021 was 1009 per 100,000 person-years, with consistently higher rates for men compared with women and a stable sex difference during the study period (2012: 18% and 2021: 20%). An overall decreasing trend in ASIR was observed during the study period, with a strong decrease from 2012 to 2017, followed by a less pronounced decline from 2018 to 2021 (Fig. 1). For men, the ASIR decreased by 32.4% in the pre-pandemic period and by 34.3% for the whole study period, and for women, ASIR decreased by 34.9% in the pre-pandemic period and by 35.7% for the whole study period. This corresponds to an average annual reduction for men and women of 5.4% and 5.9%, respectively, in the pre-pandemic period, and of 4.6% and 4.8% for the whole study period. Peaks of quarterly ASIR can be observed each year in quarter four and a subsequent drop in ASIR from quarter one to quarter three from 2012 to 2021 for both sexes, but more pronounced in men (Fig. 1). Age-stratified incidence rates show the same downward trend for those aged 50+ years, while the younger age groups 20–29, 30–39, and 40–49 show a stable trend for both sexes (Fig. 2). CIR increased with age, with the highest CIR observed for the age group 60–69 years for men and 70–79 years for women (Fig. 2).

### Regression model prediction of incidence rate of type 2 diabetes: pandemic effect

**Fig. 3:**
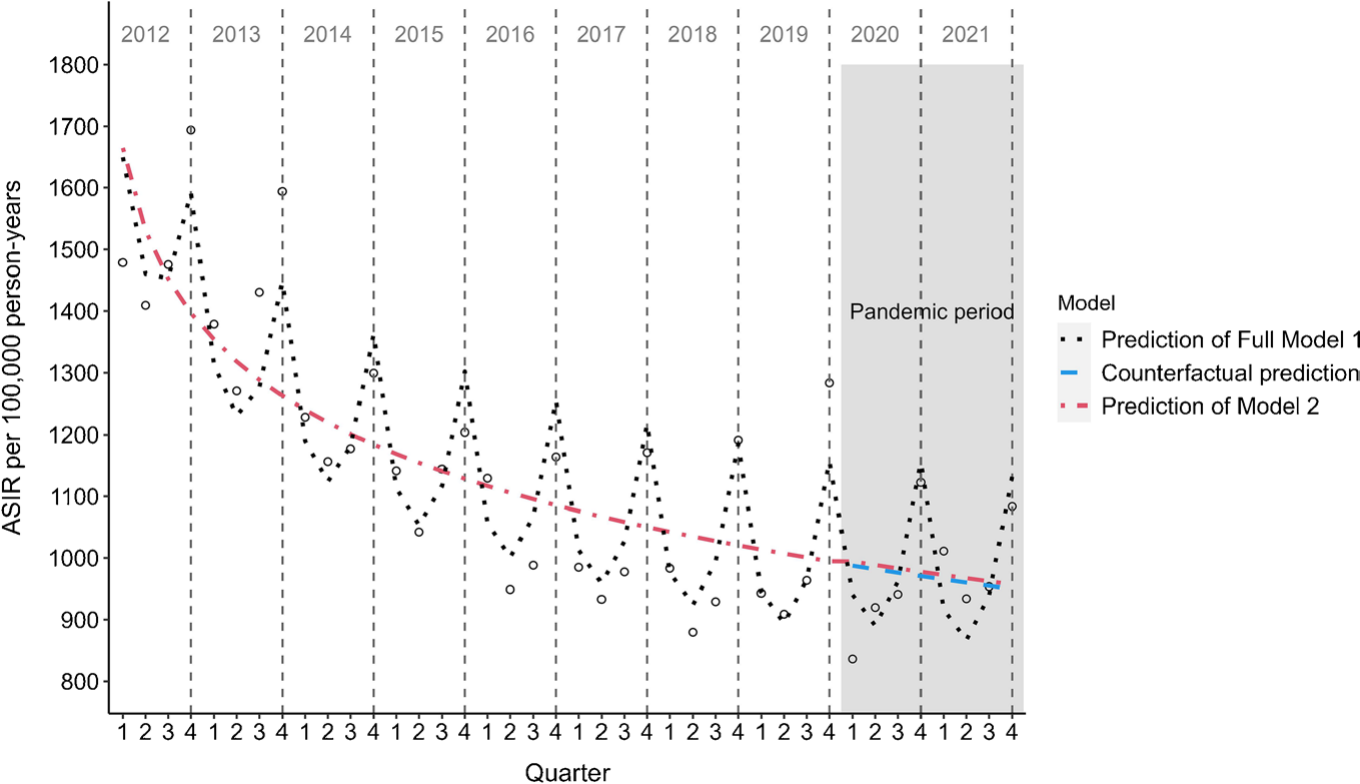
Prediction of type 2 diabetes ASIR from 2012 to 2021 for men. Black dotted line, full Model 1 adjusted for time trend, pandemic effect and seasonal effects; blue dotted line, counterfactual prediction based on Model 3 adjusted for time trend using data from 2012 to 2019; red dotted line, Model 2 adjusted for time trend and pandemic effect but without seasonal effects

**Fig. 4:**
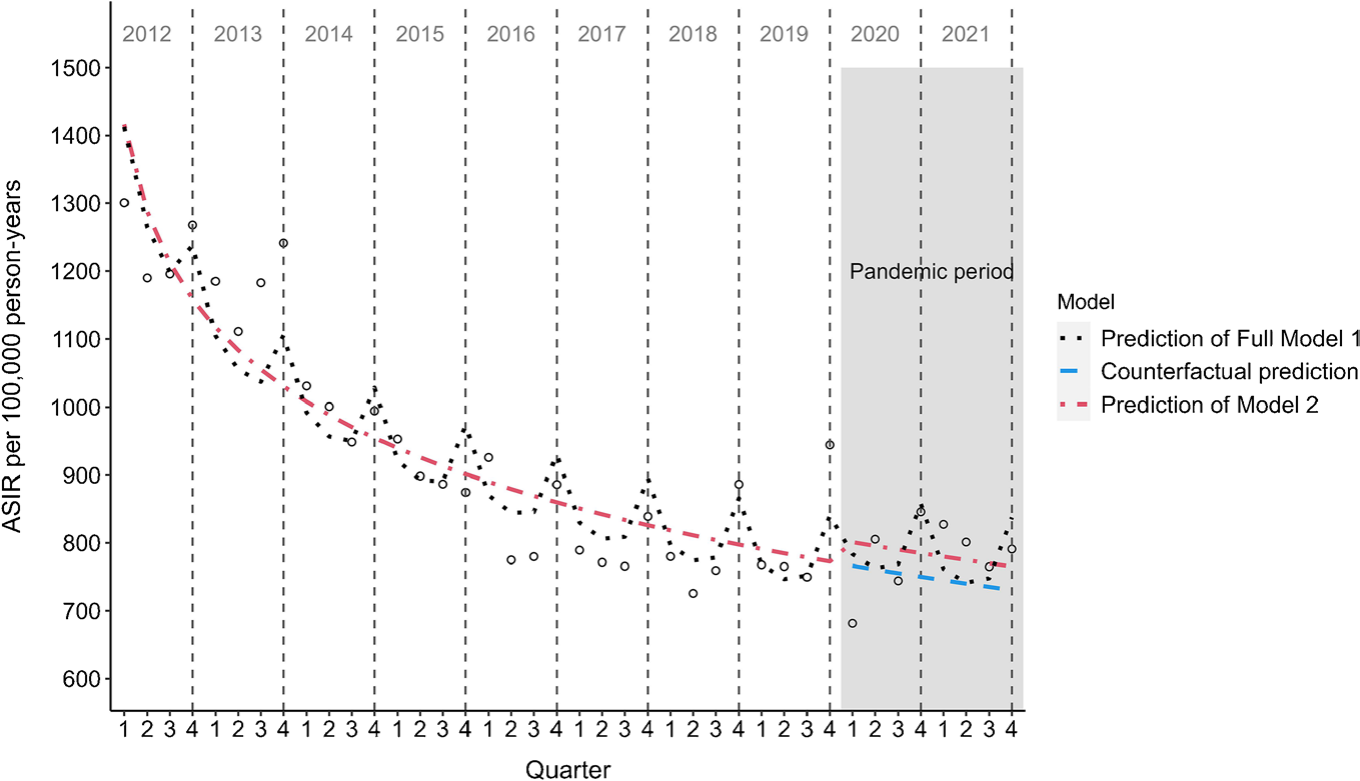
Prediction of type 2 diabetes ASIR from 2012 to 2021 for women. Black dotted line, full Model 1 adjusted for time trend, pandemic effect and seasonal effects; blue dotted line, counterfactual prediction based on Model 3 adjusted for time trend using data from 2012 to 2019; red dotted line, Model 2 adjusted for time trend and pandemic effect but without seasonal effects

Regression analyses for men using the full Model 1 show a logarithmically decreasing time trend of type 2 diabetes incidence (ß, −209.1, 95% CI −240.3, −177.8) with no significant changes in ASIR of type 2 diabetes during the pandemic period (2020–2021) when compared with the pre-pandemic period (ß, 22.1, 95% CI −44.7, 88.9) (Fig. 3). Regression analyses for women with the full Model 1 also show a logarithmically decreasing time trend of type 2 diabetes incidence (ß, −191.3, 95% CI −218.3, −164.2) with no significant changes in ASIR for type 2 diabetes during the pandemic period (2020–2021) as compared with the pre-pandemic period (ß, 40.0, 95% CI −17.8, 97.7) (Fig. 4). Regression analyses also show no relevant difference in the ASIR of type 2 diabetes between predictions of ASIR based on Model 2 (using data from 2012 to 2021) and the counterfactual predictions of ASIR based on Model 3 (using data from 2012 to 2019) for both sexes (Figs 3 and 4). Seasonal effects can be observed for both sexes with consistently lower ASIR in quarter one to quarter three compared with quarter four each year (electronic supplementary material [ESM] Table 1). The full Model 1 was significant for both men (F [5;34]=54.4, *p*<0.05) and women (F [2;37]=81.5, *p*<0.05), explaining around 87.4% of the variance in ASIR for type 2 diabetes in men and 79.8% of the variance in ASIR for type 2 diabetes in women.

### Sensitivity analyses

To account for possible misclassification of type 2 diabetes E11 ‘Diabetes mellitus, Type 2’ and E14 ‘Unspecified diabetes mellitus’, a sensitivity analysis was conducted that included E11 cases only. Overall, 686,955 cases (58,906 fewer cases) were included in the analysis, showing no differences in the time trend of type 2 diabetes incidence and no changes in the incidence rate during the pandemic period (2020–2021) compared with the pre-pandemic period (2012–2019). Further, for completeness of the regression analysis, Model 4 without pandemic effects but with seasonal effects (counterfactual prediction with seasonal effects) was used to predict the ASIR of type 2 diabetes for both sexes and identified no change in incidence rate during the pandemic (ESM Fig. 1, ESM Fig. 2).

## Discussion

Using a large aggregated health claims database, four key findings on the incidence of type 2 diabetes incidence in Bavaria, the largest German state, could be obtained. First, a decreasing incidence trend from 2012 to 2021, with a strong decrease from 2012 until 2017 followed by a less pronounced decline from 2018 to 2021, was observed for both sexes. Sex-specific ASIR decreased by 34.3% in men and by 35.7% in women from 2012 to 2021. Second, this downward trend was also observed for all 10-year age groups between 50 and 90 years, while the younger age groups 20–29, 30–39 and 40–49 years remained stable throughout for both sexes. Third, the median age at diagnosis decreased by 2.6 years for men and 4.7 years for women. Fourth, the incidence rate during the COVID-19 pandemic period (2020–2021) showed no change compared with the pre-pandemic period. This suggests that the incidence of type 2 diabetes was not affected by the first 2 years of the pandemic.

Our results correspond with previous international findings. A systematic review reporting type 2 diabetes incidence on a global level for the first time also revealed decreasing (36% of studies) or stable (30% of studies) incidence trends for most of the included high- and middle-income countries since 2010 [17, 18]. Of these, a higher proportion of the examined populations came from European countries (52%) than from non-European countries (41%) [17]. Compared with previous decades (starting in the 1960s) with increasing rates, a shift of direction in type 2 diabetes incidence trend in mainly European countries has occurred [17]. By additionally covering the pandemic period in our study, the observed decreasing incidence trend in Bavaria, Germany, not only aligns with these recent findings but allows for further assessment of the pandemic effect. In contrast to these recent international findings, a systematic analysis of the Global Burden of Disease Study 2019, which included 204 countries and territories, found an increase in type 2 diabetes incidence globally from 184.6 per 100,000 population in 1990 to 259.9 per 100,000 population in 2019 [19]. A recent study on incidence trends in Germany based on nationwide health claims data supports our results, also observing a decrease in incidence trend from 2014 to 2019 by 2.4% for women and 1.7% for men, with lower rates of 8.4 for men and 6.9 per 1000 people for women in 2014 and 7.7 for men and 6.1 per 1000 people for women in 2019 [20]. However, this study represents a short time period of 6 years and does not cover the pandemic period. Another recent study in Germany reported a decreasing type 2 diabetes incidence rate of 0.68% in 2015 and 0.57% in 2020, with slightly increased rates of 0.87% in 2021, but did not use any explanatory analysis to quantify the observed trend [21]. Goffrier et al, who also used health claims data, support our findings, reporting a nationwide 5% decrease in incidence in Germany over a short period of 3 years from 2012 to 2014. This study shows comparable incidence rates of 1630 per 100,000 people in 2012, 1600 per 100,000 people in 2013, and 1470 per 100,000 people in 2014 [22]. Further studies reporting incidence rates for Germany are either limited to very short periods, up to a maximum of 3 years, or have data based on selected health insurance funds, not allowing for any adequate inferences about the actual trend [22–25]. Most studies report incidence rates for the period before 2010, not representing current data [23–25]. Usually, slightly more men than women are affected by type 2 diabetes [26]; however, we reported an almost equal number of cases for men (50.4%) and women (49.6%) in our study. While recent prevalence data showed that more men (41.3%) are overweight than women (27.6%), obesity prevalence shows no difference in sex in Germany [27].

The downward trend of incidence observed could be due to robustness of preventive measures. A decrease in type 2 diabetes risk could be observed in Germany from 1997 to 1999 and 2008 to 2011, with the reductions in the consumption of red meat and waist circumference suggested as the main drivers [28]. However, meat consumption remained relatively stable from 2004 to 2014/2015 [29], as did the prevalence of overweight from 2012 to 2020 in Germany [27]. For most risk factors of type 2 diabetes, no major changes in the trend could be observed. While the prevalence of smoking decreased from 1995 to 2018 [30], the prevalence of obesity increased from 2012 to 2020 [27]. Also, physical inactivity levels are high in Germany [31]. Less than half of the adults (51.2% men and 44.8% women) reached the WHO recommendation of at least 2.5 h of aerobic physical activity per week before the pandemic [31] and 24% of adults reported reducing physical activity during the pandemic period in 2021 [32]. A potential reason for the decline in diabetes incidence might be sufficient screening efforts over the last 10 years. Studies show that people with screening-detected diabetes are diagnosed 3.3 to 4.6 years earlier than clinical-based detection [33, 34]. The observed decrease in the median age of diagnosis of 2.6 years for men and 4.7 years for women in our study supports this hypothesis of more effective screening by family physicians. Therefore, screening for type 2 diabetes seems to be an important step in diabetes prevention since cost-effective interventions (pharmacological and behavioural interventions) show benefits in preventing or delaying the progression of diabetes [35]. Glucose-lowering medications have been shown to improve all-cause and diabetes-related mortality for newly diagnosed type 2 diabetes compared to conventional treatment with diet [35]. Additionally, lifestyle interventions also reduce the progression of diabetes [35, 36]. Trials showed that lifestyle intervention in people with impaired glucose tolerance or impaired fasting glycaemia effectively reduced the incidence of diabetes by 28–58% compared with control groups over a period of 3 to 6 years [36].

Recent studies have hypothesised about the impact of COVID-19 on type 2 diabetes. Either a COVID-19 infection itself [37] or disease containment measures possibly altering the impact of lifestyle factors such as physical activity may increase the risk of type 2 diabetes by affecting glycaemic control and thus lead to an increase in type 2 diabetes [38, 39]. With no relevant changes in incidence in the first 2 years of the pandemic, our data suggest that patients continued to seek healthcare from their family physicians (and to a lesser extent with their specialists) [40]. However, type 2 diabetes often develops slowly over the course of several years [41], delaying the direct impact of the pandemic on incidence. Therefore, future monitoring is necessary to assess the long-term effect of the pandemic on diabetes incidence.

To accommodate the need for adequate diabetes monitoring, the German Diabetes Society has proposed a national diabetes registry [42]. Existing studies about diabetes development mainly focus on monitoring prevalence alone, with incidence rates rarely reported [1]. But incidence should also become a standard reporting metric for diabetes, since unlike prevalence, it allows the direct assessment of the effects of changes in risk factors, as it is not influenced by other factors, such as survival [17]. Recent prediction of the prevalence of type 2 diabetes in Germany projects an increase of 54–77% by 2040, with the incidence trend being the key factor in this increase [4]. Increased prevalence can coexist with decreasing incidence [20]. By monitoring and reporting both prevalence and incidence, a better understanding of the burden of type 2 diabetes can be achieved.

### Strengths and limitations

This study is the first to report the current 10-year incidence trend of type 2 diabetes for adults (>20 years) in Germany, effectively filling an important gap in diabetes monitoring, and providing a better understanding of the disease burden. Routinely collected health claims data were used in our analyses, encompassing all statutorily insured people in the state of Bavaria, with an insurance population of 11 million residents. These data represent a reliable data source and provide high accuracy for describing the incidence trend in Bavaria, the largest German state. In contrast to previous incidence studies of type 2 diabetes in Germany, which are mainly based on data from selected health insurance companies and short observation time periods [22–24], our study allows for a more extended analysis over 10 years with a complete regional sample. However, the data do not include the privately insured and typically high-income population (10–15% of the Bavarian population), who may have lower rates of diabetes than the general population [43]. Given the small share of this group of insurees in the total population, the present observed incidence rates may slightly overestimate population incidence. Additionally, this is one of the first studies reporting type 2 diabetes incidence in adults during the pandemic and comparing it to the pre-pandemic period in Germany. Only one study with a similar time period also showed decreasing incidence rates until 2020 with slightly increasing rates in 2021 in Germany [21].

General weaknesses of health claims data should be considered when interpreting the results [44]. Since claims data exist primarily for billing and reimbursement purposes, data might not always necessarily reflect just the medical burden, but also the coding and clinical practice of practitioners. This may also partly explain the seasonal patterns observed in the data. Studies investigating the seasonal patterns of the incidence of type 2 diabetes and changes in glycaemic control are inconsistent and can only partially explain the peaks in quarter four. Factors influencing seasonality include physical activity, dietary habits and ambient temperature [45–47]. In England, peak consultation occurred during hot weather waves [48], whereas in Hungary, incidence of type 2 diabetes was highest in March and lowest in August [46]. Additionally, physicians’ diagnostic coding errors could lead to misdiagnosis. To reduce any bias due to diagnostic accuracy, only cases with confirmed new diagnoses with the same ICD code twice in four consecutive quarters were included. Possible misclassification of type 2 diabetes was addressed in the sensitivity analyses. Additionally, the pandemic period only covers the first 2 years, 2020 and 2021. To investigate the later years of this global health emergency, data covering 2022 should be included as soon as reliable data are available. In contrast to quarterly data, weekly or monthly data during the pandemic could provide more information in relation to disease containment measures during the pandemic, similar to other studies primarily investigating the pandemic effect [49, 50].

### Conclusion

To fully understand the epidemic of type 2 diabetes, reporting of incidence is crucial. Although prevalence seems to increase overall, a decreasing incidence trend in Germany from 2012 to 2021 could be observed in our study. This indicates a break in the upward trajectory of disease burden, potentially resulting from better screening efforts by the family physicians and thereby reducing the median age of diagnosis by 3–5 years for both sexes. No changes in incidence for the pandemic period in 2020 and 2021 relative to the pre-pandemic period were observed, indicating that this group of patients continued to seek healthcare services even during an extraordinary healthcare crisis. Reasons for the declining incidence trend need further investigation using appropriate data sources, and continuous monitoring of the incidence is necessary to identify the long-term trend and potential effects of the pandemic on diagnoses.

## Supplementary Information

Below is the link to the electronic supplementary material.

Supplementary file1 (PDF 363 KB)

## Data Availability

The data on which these analyses were conducted are available from the KVB, but restrictions apply to the availability of these data. Data are, however, available from the authors upon reasonable request and with permission of the KVB.

## Abbreviations

ASIR: Age-standardised incidence rate
CIR: Crude incidence rate
ICD-10-GM: International statistical classification of disease, 10th revision, German modification
KVB: Bavarian Association of Statutory Health Insurance Physicians

## References

[1] International Diabetes Federation (2021) IDF Diabetes Atlas, 10th edn. International Diabetes Federation, Brussels, Belgium

[2] Saeedi P, Petersohn I, Salpea P et al (2019) Global and regional diabetes prevalence estimates for 2019 and projections for 2030 and 2045: results from the International Diabetes Federation Diabetes Atlas, 9(th) edition. Diabetes Res Clin Pract 157:107843. 10.1016/j.diabres.2019.107843 31518657 10.1016/j.diabres.2019.107843

[3] Heidemann C, Scheidt-Nave C (2017) Prevalence, incidence and mortality of diabetes mellitus in adults in Germany – a review in the framework of the Diabetes Surveillance. J Health Monit 2(3):98–121. 10.17886/rki-gbe-2017-062 37168946 10.17886/RKI-GBE-2017-062 PMC10165910

[4] Tönnies T, Röckl S, Hoyer A et al (2019) Projected number of people with diagnosed type 2 diabetes in Germany in 2040. Diabet Med 36(10):1217–1225. 10.1111/dme.13902 30659656 10.1111/dme.13902

[5] Jacobs E, Hoyer A, Brinks R, Kuss O, Rathmann W (2017) Burden of mortality attributable to diagnosed diabetes: a nationwide analysis based on claims data from 65 million people in Germany. Diabetes Care 40(12):1703–1709. 10.2337/dc17-0954 28993421 10.2337/dc17-0954

[6] Ulrich S, Holle R, Wacker M et al (2016) Cost burden of type 2 diabetes in Germany: results from the population-based KORA studies. BMJ Open 6(11):e012527. 10.1136/bmjopen-2016-012527 27872118 10.1136/bmjopen-2016-012527 PMC5129071

[7] Bak JCG, Serné EH, Kramer MHH, Nieuwdorp M, Verheugt CL (2021) National diabetes registries: do they make a difference? Acta Diabetol 58(3):267–278. 10.1007/s00592-020-01576-8 32770407 10.1007/s00592-020-01576-8 PMC7907019

[8] Epping J, Geyer S, Eberhard S, Tetzlaff J (2021) Completely different or quite similar? The sociodemographic structure of the AOK Lower Saxony in comparison to the general and working population in Lower Saxony and the Federal Republic of Germany. Gesundheitswesen 83(S 02):S77-s86. 10.1055/a-1553-3565 34695865 10.1055/a-1553-3565

[9] German Federal Ministry of Health KM 6 statistics. Available from https://www.bundesgesundheitsministerium.de/themen/krankenversicherung/zahlen-und-fakten-zur-krankenversicherung/mitglieder-und-versicherte.html. Accessed 28 August 2023

[10] Swart E, Gothe H, Geyer S et al (2015) Good Practice of Secondary Data Analysis (GPS): guidelines and recommendations. Gesundheitswesen 77(2):120–126. 10.1055/s-0034-1396815 25622207 10.1055/s-0034-1396815

[11] Deutsches Institut für Medizinische Dokumentation und Information (2020) The International Statistical Classification Of Diseases And Related Health Problems, 10th revision, German Modification. Available from https://www.dimdi.de/static/de/klassifikationen/icd/icd-10-gm/kode-suche/htmlgm2020/. Accessed November 11 2023

[12] Pace M, Lanzieri G, Glickman M et al (2013) Revision of the European standard population. Eurostat Methodologies and Working Papers. 10.2785/11470

[13] Bernal JL, Cummins S, Gasparrini A (2017) Interrupted time series regression for the evaluation of public health interventions: a tutorial. Int J Epidemiol 46(1):348–355. 10.1093/ije/dyw098 27283160 10.1093/ije/dyw098 PMC5407170

[14] R Core Team (2022) R: a language and environment for statistical computing. Available from https://www.R-project.org/. Accessed April 25 2023

[15] Wickham H, Averick M, Bryan J et al (2019) Welcome to the tidyverse. J Open Source Softw 4(43):1686. 10.21105/joss.01686

[16] Wickham H (2016) ggplot2: elegant graphics for data analysis. Springer-Verlag, New York

[17] Magliano DJ, Islam RM, Barr ELM et al (2019) Trends in incidence of total or type 2 diabetes: systematic review. BMJ 366:l5003. 10.1136/bmj.l5003 31511236 10.1136/bmj.l5003 PMC6737490

[18] Magliano DJ, Chen L, Islam RM et al (2021) Trends in the incidence of diagnosed diabetes: a multicountry analysis of aggregate data from 22 million diagnoses in high-income and middle-income settings. Lancet Diabetes Endocrinol 9(4):203–211. 10.1016/s2213-8587(20)30402-2 33636102 10.1016/S2213-8587(20)30402-2 PMC10984526

[19] Ye J, Wu Y, Yang S et al (2023) The global, regional and national burden of type 2 diabetes mellitus in the past, present and future: a systematic analysis of the Global Burden of Disease Study 2019. Front Endocrinol (Lausanne) 14:1192629. 10.3389/fendo.2023.1192629 37522116 10.3389/fendo.2023.1192629 PMC10376703

[20] Tönnies T, Hoyer A, Brinks R, Kuss O, Hering R, Schulz M (2023) Spatio-temporal trends in the incidence of type 2 diabetes in Germany. Analysis of the claims data of 63 million persons with statutory health insurance from 2014 to 2019. Dtsch Arztebl Int 120(11):173–179. 10.3238/arztebl.m2022.0405 10.3238/arztebl.m2022.0405 PMC10213473 36647586

[21] Reitzle L, Heidemann C, Jacob J, Pawlowska-Phelan D, Ludwig M, Scheidt-Nave C (2023) Inzidenz von Typ-1- und Typ-2-Diabetes vor und während der COVID-19-Pandemie in Deutschland: Analyse von Routinedaten der Jahre 2015 bis 2021. J Health Monit 8(S5):2–26. 10.25646/11703 [in German] 10.25646/11730 PMC10698802 38074488

[22] Goffrier B, Schulz M, Bätzing J (2017) Administrative Prävalenzen und Inzidenzen des Diabetes mellitus von 2009 bis 2015. Zentralinstitut für die kassenärztliche Versorgung in Deutschland (Zi). Versorgungsatlas-Bericht Nr. 17/03. Berlin 2017. 10.20364/VA-17.03 [in German]

[23] Tamayo T, Brinks R, Hoyer A, Kuß O, Rathmann W (2016) The prevalence and incidence of diabetes in Germany: an analysis of statutory health insurance data on 65 million individuals from the years 2009 and 2010. Dtsch Arztebl International 113(11):177–182. 10.3238/arztebl.2016.0177 10.3238/arztebl.2016.0177 PMC4850517 27118665

[24] Boehme MW, Buechele G, Frankenhauser-Mannuss J et al (2015) Prevalence, incidence and concomitant co-morbidities of type 2 diabetes mellitus in South Western Germany–a retrospective cohort and case control study in claims data of a large statutory health insurance. BMC Public Health 3(15):855. 10.1186/s12889-015-2188-1 10.1186/s12889-015-2188-1 PMC4559219 26334523

[25] Wilke T, Ahrendt P, Schwartz D, Linder R, Ahrens S, Verheyen F (2013) Inzidenz und Prävalenz von Diabetes mellitus Typ 2 in Deutschland. Dtsch Med Wochenschr 138(03):69–75. 10.1055/s-0032-1327394 [in German] 10.1055/s-0032-1327394 23299340

[26] Kautzky-Willer A, Leutner M, Harreiter J (2023) Sex differences in type 2 diabetes. Diabetologia 66(6):986–1002. 10.1007/s00125-023-05891-x 36897358 10.1007/s00125-023-05891-x PMC10163139

[27] Schienkiewitz A, Kuhnert R, Blume M, Mensink GBM (2022) Übergewicht und Adipositas bei Erwachsenen in Deutschland - Ergebnisse der Studie GEDA 2019/2020-EHIS. J Health Monit 7(3):23–31. 10.25646/10292 [in German]

[28] Paprott R, Mensink G, Schulze MB et al (2017) Temporal changes in predicted risk of type 2 diabetes in Germany: findings from the German Health Interview and Examination Surveys 1997–1999 and 2008–2011. BMJ Open 7(7):e013058. 10.1136/bmjopen-2016-013058 28694339 10.1136/bmjopen-2016-013058 PMC5541581

[29] German Nutrition Society (2016) 13th DGE-Nutrition Report - summary available from https://www.dge.de/fileadmin/Dokumente/WISSENSCHAFT/Ernaehrungsberichte/13-DGE-EB/DGE-Nutrition-Report-summary-2016.pdf. Accessed May 25 2023

[30] Seitz NN, Lochbühler K, Atzendorf J, Rauschert C, Pfeiffer-Gerschel T, Kraus L (2019) Trends in substance use and related disorders: analysis of the epidemiological survey of substance abuse 1995 to 2018. Dtsch Arztebl Int 116(35–36):585–591. 10.3238/arztebl.2019.0585 31587706 10.3238/arztebl.2019.0585 PMC6804271

[31] Richter A, Schienkiwitz A, Starker A et al (2021) Gesundheitsfördernde Verhaltensweisen bei Erwachsenen in Deutschland – Ergebnisse der Studie GEDA 2019/2020-EHIS. J Health Monit 6(3):28–48. 10.25646/8460. [in German]

[32] Manz K, Krug S (2022) Veränderung des Sporttreibens und der aktiven Wegstrecken seit der COVID-19-Pandemie – Ergebnisse der Studie GEDA 2021. J Health Monit 7(4):24–38. 10.25646/10665

[33] Feldman AL, Griffin SJ, Fhärm E et al (2017) Screening for type 2 diabetes: do screen-detected cases fare better? Diabetologia 60(11):2200–2209. 10.1007/s00125-017-4402-4 28831538 10.1007/s00125-017-4402-4 PMC6086324

[34] Rahman M, Simmons RK, Hennings SH, Wareham NJ, Griffin SJ (2012) How much does screening bring forward the diagnosis of type 2 diabetes and reduce complications? Twelve year follow-up of the Ely cohort. Diabetologia 55(6):1651–1659. 10.1007/s00125-011-2441-9 22237689 10.1007/s00125-011-2441-9

[35] Davidson KW, Barry MJ, Mangione CM et al (2021) Screening for prediabetes and type 2 diabetes: US preventive services task force recommendation statement. JAMA 326(8):736–743. 10.1001/jama.2021.12531 34427594 10.1001/jama.2021.12531

[36] Echouffo-Tcheugui JB, Selvin E (2021) Prediabetes and what it means: the epidemiological evidence. Annu Rev Public Health 42:59–77. 10.1146/annurev-publhealth-090419-102644 33355476 10.1146/annurev-publhealth-090419-102644 PMC8026645

[37] Rathmann W, Kuss O, Kostev K (2022) Incidence of newly diagnosed diabetes after Covid-19. Diabetologia 65(6):949–954. 10.1007/s00125-022-05670-0 35292829 10.1007/s00125-022-05670-0 PMC8923743

[38] Marçal IR, Fernandes B, Viana AA, Ciolac EG (2020) The urgent need for recommending physical activity for the management of diabetes during and beyond COVID-19 outbreak. Front Endocrinol (Lausanne) 11:584642. 10.3389/fendo.2020.584642 33250859 10.3389/fendo.2020.584642 PMC7673403

[39] Eberle C, Stichling S (2021) Impact of COVID-19 lockdown on glycemic control in patients with type 1 and type 2 diabetes mellitus: a systematic review. Diabetol Metab Syndr 13(1):95. 10.1186/s13098-021-00705-9 34493317 10.1186/s13098-021-00705-9 PMC8423337

[40] Damerow S, Rommel A, Prütz F et al (2020) Die gesundheitliche Lage in Deutschland in der Anfangsphase der COVID-19-Pandemie. Zeitliche Entwicklung ausgewählter Indikatoren der Studie GEDA 2019/2020-EHIS. J Health Monit 5(4):3–22. 10.25646/7171.2 [in German]

[41] Chatterjee S, Khunti K, Davies MJ (2017) Type 2 diabetes. Lancet 389(10085):2239–2251. 10.1016/s0140-6736(17)30058-2 28190580 10.1016/S0140-6736(17)30058-2

[42] Deutsche Ärzteblatt (2018) Diabetologen wollen nationales Diabetesregister aufbauen. Available from https://www.aerzteblatt.de/nachrichten/95129/Diabetologen-wollen-nationales-Diabetesregister-aufbauen. Accessed May 30 2023 [in German]

[43] Stauder J, Kossow T (2017) Selection or better service – why are those with private health insurance healthier than those covered by the public insurance system? Gesundheitswesen 79(03):181–187. 10.1055/s-0042-104583 27171730 10.1055/s-0042-104583

[44] Swart E, Ihle P, Gothe H, Matusiewicz D (2014) Routinedaten im Gesundheitswesen - Handbuch Sekundärdatenanalyse: Grundlagen, Methoden und Perspektiven. Hans Huber, Bern [in German]

[45] Sakamoto M, Matsutani D, Minato S et al (2019) Seasonal variations in the achievement of guideline targets for HbA(1c), blood pressure, and cholesterol among patients with type 2 diabetes: a nationwide population-based study (ABC study: JDDM49). Diabetes Care 42(5):816–823. 10.2337/dc18-1953 30739885 10.2337/dc18-1953

[46] Doró P, Benko R, Matuz M, Soós G (2006) Seasonality in the incidence of type 2 diabetes: a population-based study. Diabetes Care 29(1):173. 10.2337/diacare.29.01.06.dc05-1839 16373925

[47] Raphael A, Friger M, Biderman A (2021) Seasonal variations in HbA1c among type 2 diabetes patients on a semi-arid climate between the years 2005–2015. Prim Care Diabetes 15(3):502–506. 10.1016/j.pcd.2020.11.013 33309124 10.1016/j.pcd.2020.11.013

[48] Hajat S, Haines A, Sarran C, Sharma A, Bates C, Fleming LE (2017) The effect of ambient temperature on type-2-diabetes: case-crossover analysis of 4+ million GP consultations across England. Environ Health 16(1):73. 10.1186/s12940-017-0284-7 28701216 10.1186/s12940-017-0284-7 PMC5506566

[49] Carr MJ, Wright AK, Leelarathna L et al (2022) Impact of COVID-19 restrictions on diabetes health checks and prescribing for people with type 2 diabetes: a UK-wide cohort study involving 618 161 people in primary care. BMJ Qual Saf 31(7):503–514. 10.1136/bmjqs-2021-013613 34642228 10.1136/bmjqs-2021-013613 PMC8520602

[50] Carr MJ, Wright AK, Leelarathna L et al (2021) Impact of COVID-19 on diagnoses, monitoring, and mortality in people with type 2 diabetes in the UK. Lancet Diabetes Endocrinol 9(7):413–415. 10.1016/s2213-8587(21)00116-9 33989537 10.1016/S2213-8587(21)00116-9 PMC8112824

