# Correction: Incidence trend of type 2 diabetes from 2012 to 2021 in Germany: an analysis of health claims data of 11 million statutorily insured people

**DOI:** 10.1007/s00125-025-06460-0

**Published:** 2025-06-17

**Authors:** Carolin T. Lehner, Marian Eberl, Ewan Donnachie, Luana F. Tanaka, Gunther Schauberger, Florian Schederecker, Sebastian Himmler, Leonie Sundmacher, Stefanie J. Klug

**Affiliations:** 1 Chair of Epidemiology, School of Medicine and Health, Technical University of Munich, Munich, Germany; 2 Bavarian Association of Statutory Health Insurance Physicians, Munich, Germany; 3 Chair of Health Economics, School of Medicine and Health, Technical University of Munich, Munich, Germany


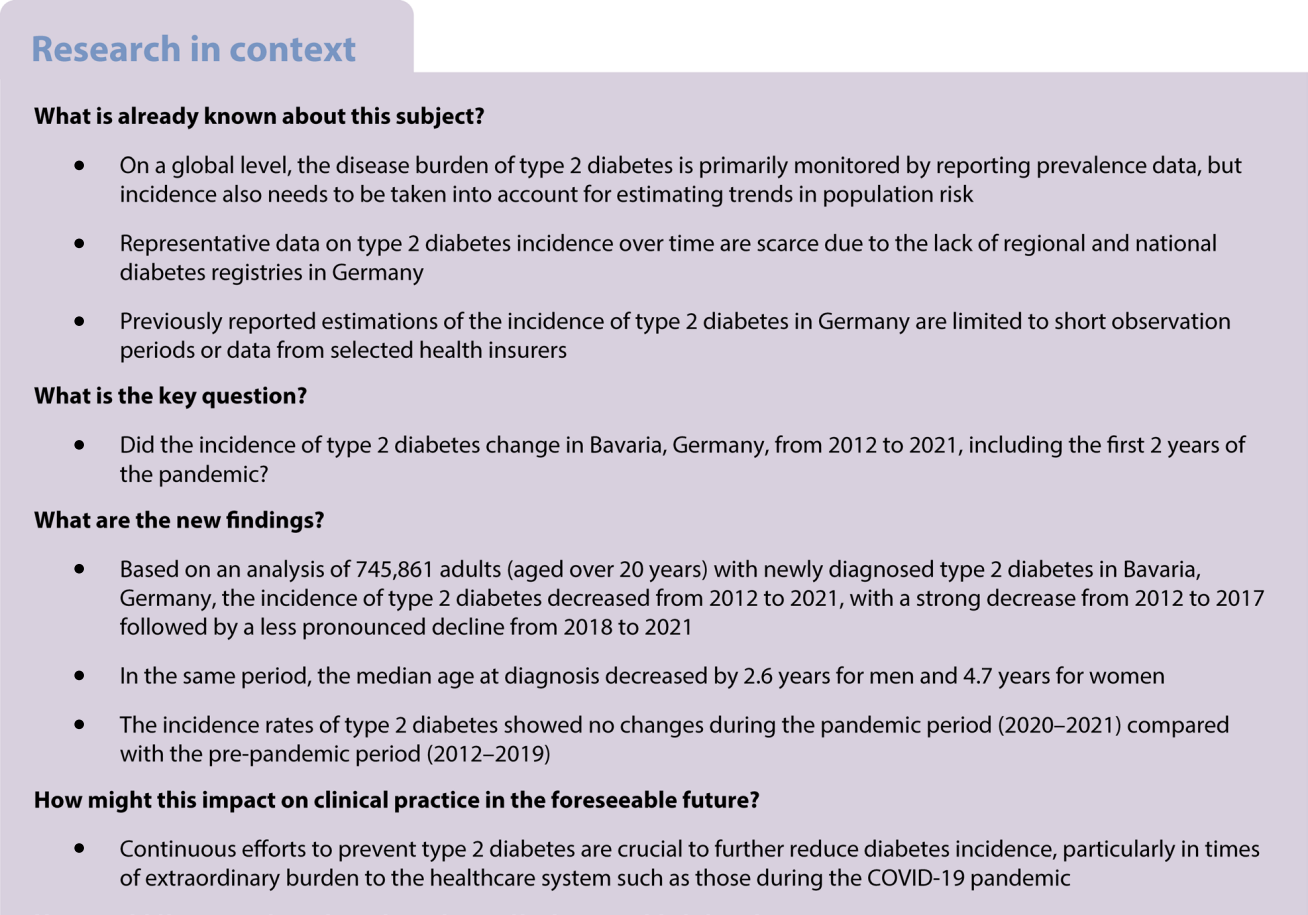


**Correction: Diabetologia**

10.1007/s00125-024-06113-8

Unfortunately, there was an error in the calculation of the median age of diagnosis, resulting in a slight underestimation of the reported numbers. The values for the median age of diagnosis have been corrected in Table 1 and the text has been updated where these data are mentioned. The original article has been corrected.

**Table 1:** Demographics of the study population aged 20 years and older

	Male					Female				
Year	New cases, (*n*)	Mean population at risk, (*n*)	CIR^a^	ASIR^b^	Median age, (years)	New cases, (*n*)	Mean population at risk, (*n*)	CIR^a^	ASIR^b^	Median age, (years)
2012	45,665	3,447,748	1324	1514	61.62	49,214	4,125,290	1192	1238	66.08
2013	43,434	3,481,866	1247	1418	61.32	47,187	4,142,374	1139	1179	65.14
2014	38,067	3,542,583	1074	1215	60.44	40,121	4,171,021	961	993	64.32
2015	35,999	3,592,838	1001	1132	62.48	36,724	4,195,541	875	903	63.55
2016	34,349	3,664,069	937	1057	59.42	34,599	4,234,879	817	841	62.86
2017	33,849	3,748,788	902	1016	59.17	32,846	4,277,835	767	791	62.60
2018	33,834	3,814,124	887	995	59.33	33,181	4,316,388	768	787	62.34
2019	35,354	3,827,483	923	1024	59.41	34,453	4,338,962	794	806	62.10
2020	33,599	3,862,386	869	954	59.16	33,186	4,366,781	759	769	61.54
2021	35,518	3,877,976	915	995	59.06	34,682	4,380,246	791	796	61.39
Total	369,668			1132	61.36	376,193			911	65.58

^a^ CIR, crude incidence rate per 100,000 person-years

^b^ ASIR, age-standardised incidence rate per 100,000 person-years using European Standard Population 2013

New cases, new incident cases of type 2 diabetes; Median age, median age at diagnosis

