# Supplementary material for: Incidence trend of type 2 diabetes from 2012 to 2021 in Germany: an analysis of health claims data of 11 million statutorily insured people
Source: Diabetologia. 2024 Feb 26;67(6):1040–50. doi: 10.1007/s00125-024-06113-8 (PMC11058936; doi:10.1007/s00125-024-06113-8)
Supplement: Supplementary file 1 — Supplementary file1 (PDF 363 KB) [file 125_2024_6113_MOESM1_ESM.pdf]

Incidence Trend of Type 2 Diabetes from 2012 to 2021 in Germany: An analysis of health claims data of 11 million statutorily insured people

Electronic supplementary material (ESM)

ESM Table 1. Regression models 1 and 2 for modeling type 2 diabetes age-standardised incidence rate by sex

	**Age-standardized incidence rate**			
	**Men**		**Women**	
	Model 1 with pandemic and seasonal effect (coefficient; 95% CI[a])	Model 2 with pandemic but without seasonal effect (coefficient; 95% CI[a])	Model 1 with pandemic and seasonal effect (coefficient; 95% CI[a])	Model 2 with pandemic but without seasonal effect (coefficient; 95% CI[a])
**Time in yearly quarters**	-209.1* (-240.3,-177.8)	-193.3* (-247.2, -139.5)	-191.3* (-218.3, -164.2)	-185.6* (-216.9, -154.2)
**Quarter 1**	-232.5* (-299.1, -166.0)	-	-91.0* (-148.6, -33.3)	-
**Quarter 2**	-276.8* (-342.9, -210.6)	-	-106.0* (-163.1, -48.8)	-
**Quarter 3**	-199.1* (-265.1, -133.2)	-	-94.5* (-151.6, -37.4)	-
**Pandemic Period**	22.1 (-44.7, 88.9)	5.6 (-110.5, 121.7)	40.0 (-17.8, 97.7)	34.0 (-33.6, 101.6)
**Constant**	1.882,0* (1.785,2, 1.978,7)	1.664,7* (1.520,2, 1,809,3)	1.503,2*	1.415,9* (1.331,8, 1,500,0)
**Corrected AIC**	-87.4	-42.1	-77.4	-58.0
**Adjusted R^2**	0.874	0.591	0.859	0.798
**F Statistic**	54.5* (df = 5; 34)	32.0* (df = 2; 37)	48.2*(df = 5; 34)	81.5* (df = 2; 37)

[a] unstandardised regression coefficients 95% confidence interval

*p<0.05

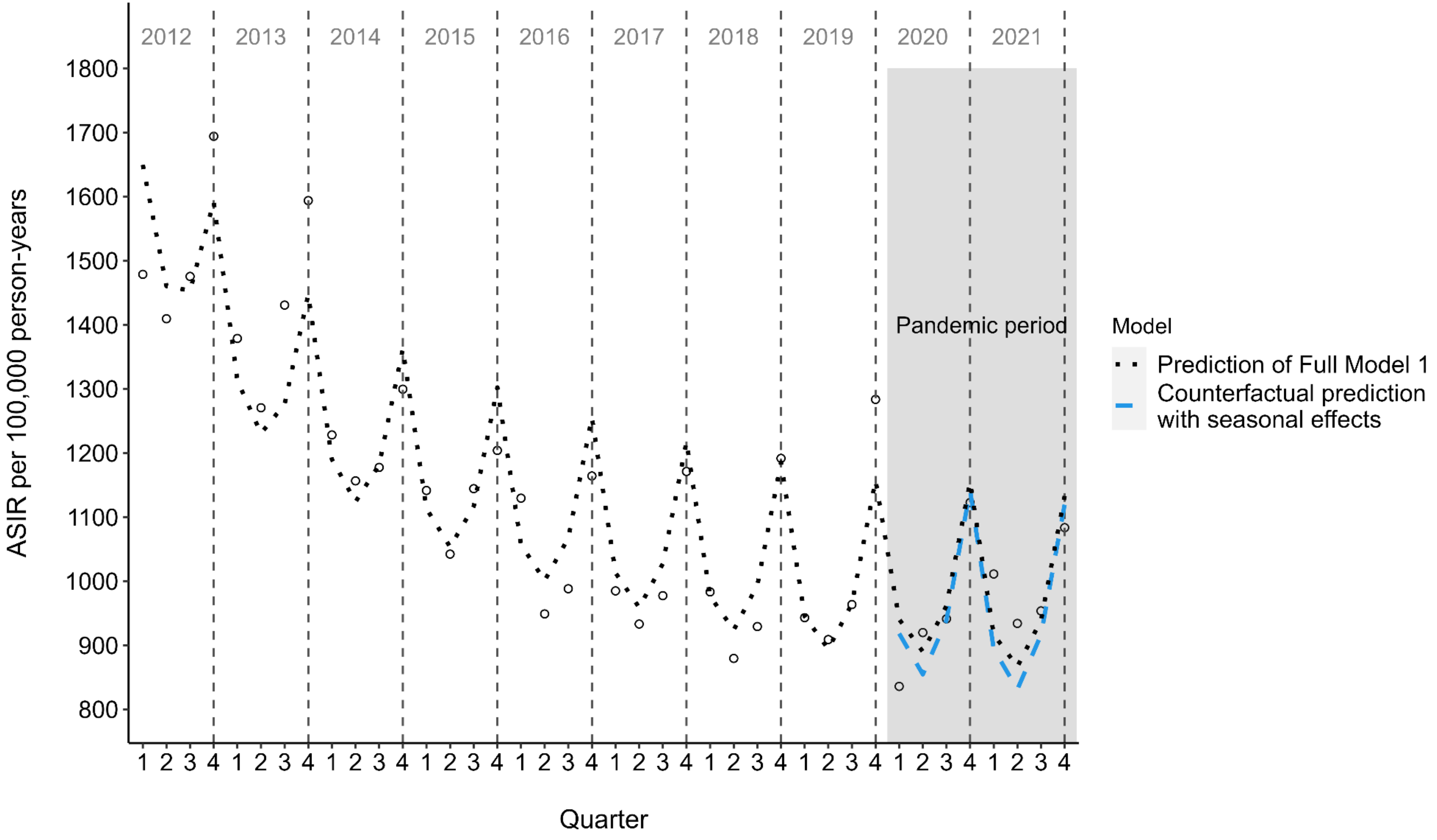

ESM Fig. 1. Prediction of type 2 diabetes ASIR from 2012 to 2021 for men. Black dotted line, full Model 1 adjusted for time trend, pandemic effect and seasonal effects; blue dotted line, predictions based on Model 4 adjusted for time trend and seasonal effects using data from 2012 to 2019 (counterfactual prediction with seasonal effects)

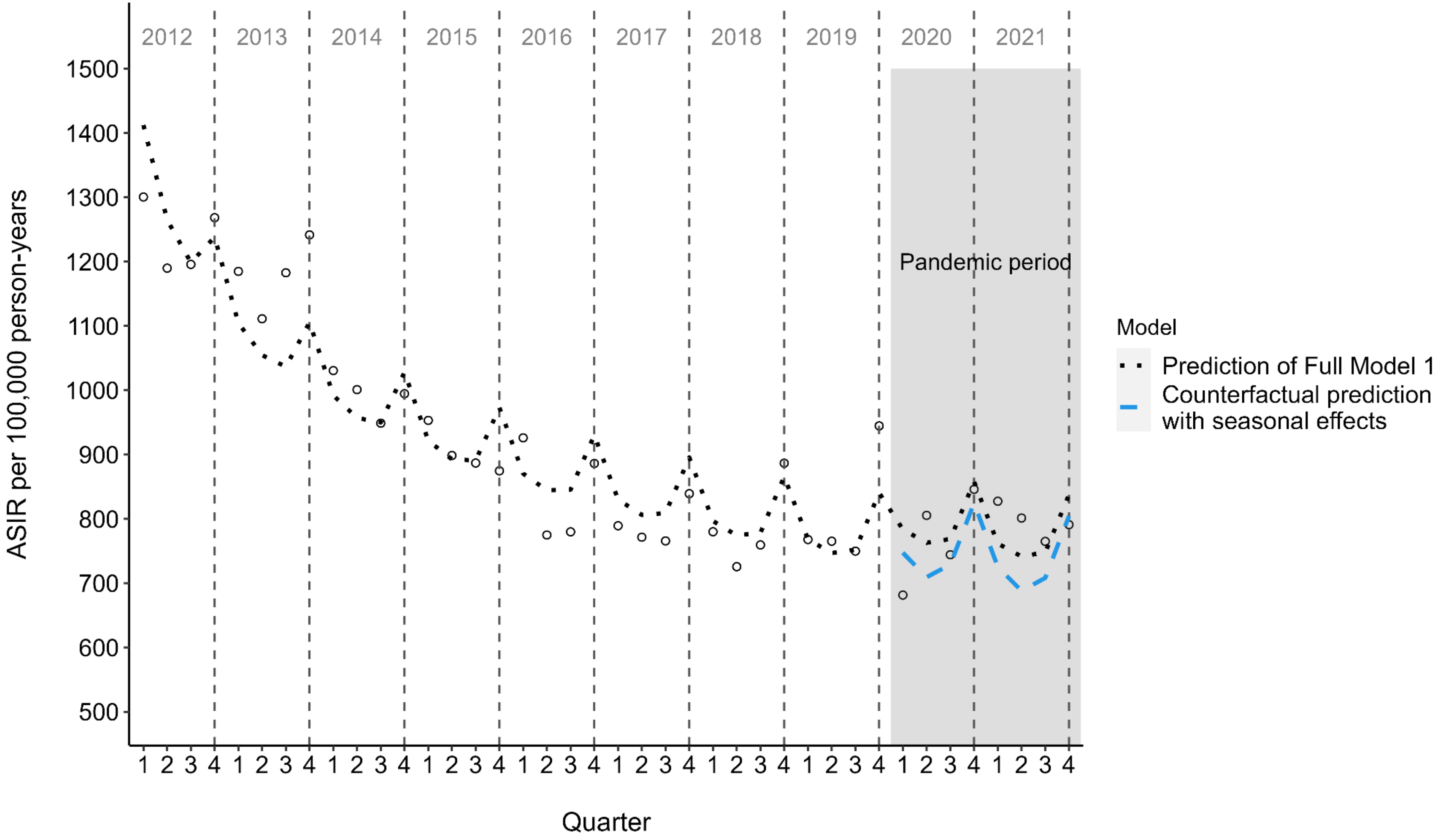

ESM Fig. 2. Prediction of type 2 diabetes ASIR from 2012 to 2021 for women. Black dotted line, full Model 1 adjusted for time trend, pandemic effect and seasonal effects; blue dotted line, predictions based on Model 4 adjusted for time trend and seasonal effects using data from 2012 to 2019 (counterfactual prediction with seasonal effects)
